# The mediating effect of discrimination, social support and hopelessness on self-rated health of Roma adolescents in Slovakia

**DOI:** 10.1186/s12939-015-0270-z

**Published:** 2015-11-19

**Authors:** Peter Kolarcik, Andrea Madarasova Geckova, Sijmen A. Reijneveld, Jitse P. Van Dijk

**Affiliations:** Graduate School Kosice Institute for Society and Health, Faculty of Medicine, P.J. Safarik University, Trieda SNP 1, 040 11 Košice, Slovakia; Department of Health Psychology, Faculty of Medicine, P.J. Safarik University, Trieda SNP 1, 040 11 Košice, Slovakia; Olomouc University Social Health Institute, Sts Cyril and Methodius Faculty of Theology, Palacky University Olomouc, Univerzitní 22, 771 11 Olomouc, Czech Republic; Department of Social Medicine, University Medical Center Groningen, University of Groningen, Antonius Deusinglaan 1, 9713 AV Groningen, The Netherlands

**Keywords:** Roma ethnicity, Adolescents, Discrimination, Social support, Hopelessness, Self-rated health

## Abstract

**Background:**

According to the EU-MIDIS report on discrimination, Roma are the most discriminated against group in Europe. Research suggests that experiencing discrimination may itself be detrimental to health. The aim of this paper is to investigate whether discrimination, hopelessness and social support mediate differences in self-rated health (SRH) between Roma and non-Roma adolescents.

**Methods:**

We conducted a cross-sectional study among Roma from separated and segregated settlements in the eastern part of Slovakia (N = 330; mean age = 14.50; interview) and non-Roma adolescents (N = 722; mean age = 14.86; questionnaire); only non-missing data were used for analyses (n = 759). The effect of perceived discrimination, mother and father social support, and hopelessness on SRH was analysed as crude and adjusted for ethnicity, age, gender, parental education and social desirability. Mediating effects were separately assessed using the Sobel test and structural equation modelling.

**Results:**

Roma adolescents reported poorer SRH and more discrimination, mother and father social support, hopelessness and social desirability. Roma ethnicity (Odds ratio/95 %-Confidence interval 3.27/2.40–4.47), discrimination (2.66/1.82–3.88), hopelessness (1.35/1.20–1.51) and mother (0.92/0.88–0.97) and father social support (0.96/0.93 – 0.997) were statistically significant predictors of poor SRH. Perceived discrimination, social support and hopelessness mediated the ethnicity-health association, with adjustment for social support increasing its strength and the other two variables decreasing it.

**Conclusions:**

Perceived discrimination, social support and hopelessness mediate a part of the association between Roma ethnicity and poor SRH, with discrimination and hopelessness being risk factors and social support a protective factor.

## Background

According to the EU-MIDIS report on discrimination, Roma are the most discriminated group in Europe, and this may still be an underestimation, as under-reporting of discrimination is generally high among Roma [1]. Stereotypes about and prejudices against Roma highly influence their status in society and lead to open and covert discrimination by the non-Roma population [2–4]. Physical attacks by right-wing extremists occur regularly and occasionally result in the death of the victim [5]. Discrimination against Roma also occurs in institutions [2]. A high percentage of Roma reports being discriminated against in health facilities, and research conducted among medical providers confirms that many of them hold prejudicial beliefs about Roma. The most frequently reported manifestations of discrimination include: general practitioners refusing to register Roma clients on their rosters, emergency services not responding to calls from Roma communities, health service providers refusing to treat them, as well as verbal abuse, denial of access to medical records and segregation into maternity wards of inferior quality [6].

Roma are a rather large minority in many Central European countries and generally report poorer health than the non-Roma population. Estimates on their numbers vary; e.g. for Slovakia, they vary from 105,738 (2.0 %) to 750,000 (13.8 %), with 380,000 (7.2 %) probably being the best estimate (2011 census) [7]. Roma adolescents have been shown to perceive their health as poorer than non-Roma adolescents [8], but the mechanisms explaining this finding have not yet been established. One explanation is that their poorer perceived health is simply due to their socioeconomic deprivation and discrimination [6, 8, 9].

Research suggests that experiencing discrimination is itself detrimental to health [10]. This concerns not just exposure but in particular some types of responses to the exposure seem to have health consequences [10]. The association between discrimination and health may occur through the mechanisms of stress responses and health behaviours. The perception of discrimination is related to increased physiological stress responses, more negative psychological stress responses and increased participation in unhealthy behaviours [11]. The negative association between discrimination and health was shown in several studies [12–15].

The poor health of Roma might also be due to factors other than discrimination, such as their on average lower socioeconomic status (SES) compared to the non-Roma population [7]. People with low SES in general perceive their health as poorer, and this association has been shown to be mediated by a number of psychosocial factors such as social support, depression, hopelessness and life satisfaction [16–20]. Self-rated Health (SRH) is a valid indicator of current health status and of future morbidity and mortality [21]. Smith et al. showed that lower SES groups have a higher prevalence of poorer SRH and a higher incidence of many predictors of poor SRH such as physical health problems, mental health problems, health care utilization, and unhealthy behaviours [22]. Another study shows that the probability of reporting poor SRH generally increased when SES became lower or neighbourhoods became more disadvantaged. This finding clearly suggests that the more life resources an individual has, the lower the risk is of reporting poor/very poor health [23]. Given the mostly low SES of Roma, this offers a rather likely explanation for their poor health as well.

In contrast to low SES, social support has a positive relation to health, but it is unknown how this association operates in Roma [24]. Among both adults and adolescents, social support – i.e. resources provided by other people, such as various social networks and relationships – is associated with positive health outcomes and the avoidance of risk-taking behaviour [24]. Klineberg et al. report ethnic differences in social support, with associations between social support and health characteristics being similar across different ethnic groups [24, 25]. Evidence on Roma is completely lacking, but social structures among them are generally strong [26].

Another factor affecting the health of Roma might be hopelessness. In general, disadvantaged people are more likely to perceive themselves as hopeless to change their situation and to improve their quality of life and well-being [27]. Such beliefs have a negative effect on health. Hopelessness correlates positively with depression, predicts suicidal ideation and attempts and psychopathology in general, and is negatively correlated with self-esteem and social skills [28]. Banks et al. found that increased levels of hope were strongly related to reporting fewer depressive symptoms when respondents reported discrimination than among those with lower levels of hope [29]. All in all, perceived discrimination obviously acts as a health-risk factor, and its negative effect might be buffered by a potential health-protective factor such as social support or magnified by the negative health effect of hopelessness.

When a sensitive topic is surveyed, and perceived discrimination might be considered as such a topic, results may be confounded by a respondent’s tendency to answer in a socially desirable way [30]. Social desirability reflects the tendency on behalf of the subjects to deny socially undesirable traits and to claim socially desirable ones, and the tendency to say things which place the speaker in a favourable light [31]. Bardwell and Dimsdale have summarised several studies that reported ethnic differences in response bias [32]. Therefore, such bias should be considered when assessing psychosocial variables by self-report.

The findings about the negative association between Roma ethnicity and their health are widely acknowledged and accepted, but further insight into the pathways leading to this association is lacking. It is also known that Roma face discrimination in everyday life, have higher levels of hopelessness, but receive more social support. All those variables are also known as significant predictors of health. Thus the aim of our study is to test the possible mediating role of those three variables on the ethnicity-health pathway and how those variables contribute to differences in self-rated health between Roma and non-Roma adolescents.

## Methods

### Sample and procedure

We obtained information on perceived discrimination, self-rated health, hopelessness, parental social support, social desirability and demographic characteristics among Roma and non-Roma adolescents. The Roma sample was recruited via elementary schools in small towns and villages in the eastern part of Slovakia which met the following criteria: the number of children aged 13 years or older living in Roma settlements (segregated and separated type) was at least 30; the school was able to provide 3 or 4 separate rooms where interviews could be conducted without disruption; and the school made an internal list of children suitable for our study, who could then be randomly chosen and asked to participate in the interview. We contacted 22 elementary schools in municipalities in the study area that had separated or segregated communities of Roma whose children could potentially attend the schools. Out of these, 15 met our criteria, though one was not willing to participate. From the lists of pupils living in Roma settlements prepared by the remaining 14 schools, we randomly chose respondents while trying to include a similar proportion of boys and girls. Respondents were interviewed individually during regular class time by community workers who had ample experience in working with Roma and were trained for our study. One hour was scheduled for each interview; they lasted between 30 and 65 minutes.

Because non-Roma pupils in schools with higher proportions of pupils from Roma settlements might not be representative of all non-Roma adolescents, we decided to recruit a non-Roma sample from elementary schools in the same geographical area without an evident Roma community in the neighbourhood. We identified 25 such schools in the Košice and Prešov regions of eastern Slovakia and contacted a random sample of 15 of them. Of these, 11 schools were willing to participate, but two were excluded because they did not have at least one class of 8^th^ and 9^th^ grade that had not been previously included in a research project of our department. The questionnaires were administered during regular class time (45 minutes) by our research assistants, who had training and experience. The questionnaire asked the same questions as the structured interview in the Roma sample.

The study was approved by the Ethics Committee of the Faculty of Science at P.J. Safarik University in Košice. Data were collected in May-June 2007. Parents were informed of the study via the school administration and could opt out if they disagreed. Participation in the study was fully voluntary and anonymous, with no explicit incentives provided for participation.

The sample of Roma adolescents consisted of 330 Roma elementary school pupils, all living in Roma settlements (the segregated and separated types) in the eastern part of Slovakia, in or near small towns and villages (response: 99.7 %). It comprised 160 boys (48.5 %) and 170 girls (51.5 %), with ages ranging from 12 to 17 years (mean 14.50; SD = 1.03). The sample of non-Roma adolescents consisted of 722 elementary school pupils attending the 8^th^ and 9^th^ grades (response 95.9 %). It comprised 354 boys (53.2 %) and 312 (46.8 %) girls. Ages ranged from 14 to 17 years (mean 14.86; SD = 0.63). High response rates were achieved due to the way of acquiring parental consent with the study, using elementary school administration.

### Measures

Questionnaires covered *demographic* (age, gender) and *socioeconomic characteristics* (father’s and mother’s highest completed education; four levels of education were distinguished: elementary education, apprenticeship, secondary education (with leaving certificate) and university education), one item assessing *self-rated health,* and scales for *social desirability*, perceived *social support* from mother and father, *hopelessness* and one item for *perceived discrimination*. All scales and items were translated from the English original to Slovak by means of a forward-backward procedure. An expert panel solved the translation issues that came out of the forward and backward translation. We did not pilot the translated scales.

Perceived discrimination was measured using an item adopted from the ISRD questionnaire [33]: “Have people ever treated you badly because of your religion or the language you speak, or the colour of your skin?” with a four-point scale ((1) No, never, (2) Once, (3) Sometimes, (4) Often). For the purposes of the analyses the four response categories were dichotomized into: No, Never (0) and at least once (1).

*Self-rated health* (SRH) was measured with one item from the SF-36 questionnaire [34]. Respondents were asked to assess their health (In general, would you say your health is:) as (1) excellent, (2) very good, (3) good, (4) fairly good or (5) bad. First two (1-2) and the last three (3-5) responses were merged into two resulting categories, similarly as was it was performed by Geckova et al., because the standard dichotomisation resulted in unbalanced categories [35]. The use of a different cut-off led to very similar results. This measure is widely used in health studies as an indicator of general health status, because it is a good predictor of mortality and morbidity [36, 37].

*Perceived social support* from the mother, father and significant others was measured using adapted items from the ‘Spouse/partner perceived social support’ subscale [38]. Items focused on aspects like closeness with the respondent, availability for chatting with the respondent, expressing worth to the respondent, feeling relaxed when together, being available when needed and confidence in the respondent. Mother’s and father’s social support subscales had 6 items, each with the following response categories (values): fully agree (4), agree (3), disagree (2), fully disagree (1). A higher total score indicates a higher level of perceived social support from the person concerned. The internal consistencies of the scales were satisfactory: mother (Cronbach’s alpha: 0.83), father (0.91).

*Hopelessness* was measured by the brief Hopelessness Scale for Children [39], which contains 5 items from the longer version of Kazdin et al. [40]. The items were: “All I see ahead of me are bad things, not good things; There’s no use in really trying to get something I want because I probably won’t get it; I might as well give up because I can’t make things better for myself; I don’t have good luck now and there’s no reason to think I will when I get older; I never get what I want, so it’s dumb to want anything.” Answers were dichotomous (values): agree (1), disagree (0), with a higher total score indicating a higher level of hopelessness. The internal consistency of the scale was satisfactory (Cronbach’s alpha: 0.70).

*Social desirability* is the tendency of respondents to reply in a manner that will be viewed favourably by others. Higher social desirability thus can affect the validity of results. It was measured using the Social Desirability Response Set (SDRS-5) [41]. The scale inquires about common situations in which people are prone to respond favourably (e.g.: “No matter who I’m talking to, I’m always a good listener“). The five items are then rated with a five-point Likert scale (definitely true, mostly true, don’t know, mostly false, definitely false). The total score is counted only from the extreme answers of each item (scored 1 point), with a higher total score indicating a higher level of socially desirable responses. Cronbach’s α for the current sample was 0.53, but the mean inter-item correlation was 0.19. According to Clark & Watson [42] and Parker, Taylor, & Bagby [43], consistency is acceptable if the MIIC is above 0.15.

### Statistical analysis

From the total number of 1052 respondents we excluded respondents who had missing answers for at least one of the assessed variables (ethnicity, age, gender, mother and father social support, hopelessness, discrimination, social desirability), leaving 759 for analysis. First, we described the samples. Then, the association of ethnicity with (poor) SRH and the way in which discrimination, social support, hopelessness, SES (highest parental education) and social desirability affected this association’, were assessed using multilevel logistic regression in order to take into account the hierarchical nature of sampling (random sample of locality at first level and then random sample of student at second level) (Table 2). As a first step, the crude associations of ethnicity, discrimination, social support, hopelessness and confounding variables (age, gender, parental education, social desirability) with SRH were assessed (Model 1, bivariate model), to see individual associations of independent variables with the dependent one. Next, we adjusted the ethnicity effect separately for perceived discrimination (Model 2), for social support (Model 3) and for hopelessness (Model 4), to see changes in the odds ratios of ethnicity regarding health outcomes after introducing these variables. Similarly, Model 5 tested the ethnicity effect adjusted for discrimination, social support, and hopelessness combined. The last model (Model 6) repeated Model 5 with adjustment for confounders: age, gender, parental education and social desirability, to consider their combined influence on the ethnicity-health association. Possible mediating effects of discrimination, hopelessness and social support on the association between ethnicity and SRH were separately assessed using the Sobel test [44]. The final mediating model was assessed using structural equation modelling. Analyses were performed using IBM SPSS 22.0 and SPSS AMOS 22.0.

## Results

The final sample is made up of 759 adolescents, of which non-Roma N = 459 (60.5 %; 234 boys = 51.0 % and 225 girls = 49.0 %) and Roma N = 300 (39.5 %; 147 boys = 49.0 %, 153 and girls = 51.0 %). Basic descriptive statistics of the sample are presented and compared in Table 1.

**Table 1:** Sociodemographic characteristics, self-rated health, discrimination, social supports, hopelessness and sensitivity for social desirability of the Roma and non-Roma samples (numbers, percentages, and p-values for differences between the two groups)

Categorical variables		Roma (N = 300)		Non-Roma (N = 459)		
		N	%	N	%	*p* value
Gender						not significant^a^
	Boys	147	49.0	234	51.0	
Father's educational level						*p* < 0.001^a^
	Elementary	154	52.6	8	1.8	
	Apprenticeship	112	38.2	86	19.9	
	Secondary	20	6.8	220	48.9	
	University	7	2.4	136	30.2	
Mother's educational level						*p* < 0.001^a^
	Elementary	215	73.9	19	4.2	
	Apprenticeship	58	19.9	68	15.0	
	Secondary	16	5.5	222	49.1	
	University	2	0.7	143	31.6	
Parents' highest educational level						*p* < 0.001^a^
	Elementary	137	45.7	5	1.1	
	Apprenticeship	127	42.3	47	10.2	
	Secondary	28	9.3	217	47.3	
	University	8	2.7	190	41.4	
Poor self-rated health		152	50.7	102	22.2	*p* < 0.001 ^a^
Discrimination		93	31.0	42	9.2	*p* < 0.001 ^a^
*Continuous variables*						
		Mean (SD)		Mean (SD)		
Age		14.5 (SD 1.0)		14.8 (SD 0.6)		*p* < 0.001^b^
Social Desirability		2.2 (SD 1.3)		1.0 (SD 1.1)		*p* < 0.001^c^
Hopelessness		1.2 (SD 1.3)		0.7 (SD 1.2)		*p* < 0.001^c^
Mother’s social support		21.2 (SD 2.6)		20.3 (SD 3.2)		*p* < 0.001^c^
Father’s social support		20.8 (SD 3.2)		18.9 (SD 4.4)		*p* < 0.001^c^

^a^Chi-square tests

^b^Student’s t-test

^c^\Mann-Whitney U-test

SD-standard deviation

Roma adolescents reported more perceived discrimination, poorer SRH as well as more mother’s and father’s social support, more hopelessness and more social desirability (Table 1). Roma came from families with parents mostly with elementary education, which reflects the poor educational level among this minority and their lower socioeconomic status.

Logistic regression showed that Roma ethnicity was a significant predictor of poor SRH. Statistically significant crude associations with SRH were also found for discrimination, hopelessness and mother’s and father’s social support. Respondents who reported being discriminated against, lower social support and higher hopelessness were more likely to report poor SRH.

The adjustment for discrimination (Model 2) and hopelessness (Model 4) led to a decrease of the ethnicity effect on SRH of about one-fifth. Adding social support (Model 3) led to an increase of the ethnicity effect on SRH of about 42 %. The adjustment for the discrimination, social support and hopelessness combined (Model 5) decreased the ethnicity effect to a smaller extent than did discrimination and hopelessness separately. Additional adjustment for the confounders (age, gender, parental education, social desirability) led to a decrease of the ethnicity-SRH effect of about one-third (Table 2).

**Table 2:** The effects of Roma ethnicity on poor self-rated health among Roma and non-Roma adolescents adjusted for discrimination, mother’s and father’s social support, and hopelessness, and adjusted for age, gender, parental education attainments and social desirability in six multilevel regression models, leading to odds ratios (OR) and 95 % confidence intervals (CI) with the locality on the second level

	Model 1	Model 2	Model 3	Model 4	Model 5	Model 6
	OR (95 % CI)	OR (95 % CI)	OR (95 % CI)	OR (95 % CI)	OR (95 % CI)	OR (95 % CI)
Roma vs. non-Roma	3.45 (2.43 – 4.87) ***	3.02 (2.12 – 4.32) ***	4.26 (2.99 – 6.06) ***	3.12 (2.17 – 4.46) ***	3.43 (2.33 – 5.05) ***	2.69 (1.51 – 4.78) **
Discrimination	2.12 (1.40 – 3.21) ***	1.89 (1.26 – 2.82) **			1.69 (1.12 – 2.55) *	1.59 (1.05 – 2.43) *
Mother‘s social support	0.89 (0.86 – 0.94) ***		0.91 (0.86 – 0.97) **		0.93 (0.87 – 0.98) *	0.93 (0.87 – 0.99) *
Father‘s social support	0.93 (0.89 – 0.97) **		0.95 (0.91 – 0.99) *		0.96 (0.91 – 1.00)	0.96 (0.92 – 1.01)
Hopelessness	1.30 (1.15 – 1.47) ***			1.26 (1.12 – 1.42) ***	1.20 (1.06 – 1.36) **	1.21 (1.07 – 1.37) **
Age in years	0.89 (0.73 – 1.09)					0.89 (0.73 – 1.09)
Gender (Male vs. Female)	0.67 (0.49 – 0.93) *					0.68 (0.49 – 0.94) *
Parental education						
Elementary	(reference) ***					(reference)
Apprenticeship	0.71 (0.45 – 1.13)					0.90 (0.55 – 1.45)
Secondary	0.33 (0.20 – 0.55) ***					0.64 (0.34 – 1.18)
University	0.23 (0.13 – 0.40) ***					0.54 (0.27 – 1.10)
Social desirability	0.92 (0.80 – 1.06)					0.89 (0.77 – 1.03)
Reduction of OR for Roma ethnicity after adjustment, compared to Model 1	---	17.55	-33.06	13.47	0.82	31.02

Model 1: Crude effect of each variable separately on self-rated health

Model 2: Effect of Roma ethnicity on self-rated health adjusted for discrimination

Model 3: Effect of Roma ethnicity on self-rated health adjusted for mother’s and father’s social support

Model 4: Effect of Roma ethnicity on self-rated health adjusted for hopelessness

Model 5 Effect of Roma ethnicity on self-rated health adjusted for discrimination, mother’s and father’s social support and hopelessness

Model 6: Effect of Roma ethnicity on self-rated health adjusted for discrimination, mother’s and father’s social support and hopelessness and controlled for age, gender, parental education, and social desirability

***p < 0.001, ** *p* < 0.01, * *p* < 0.05

**Fig. 1:**
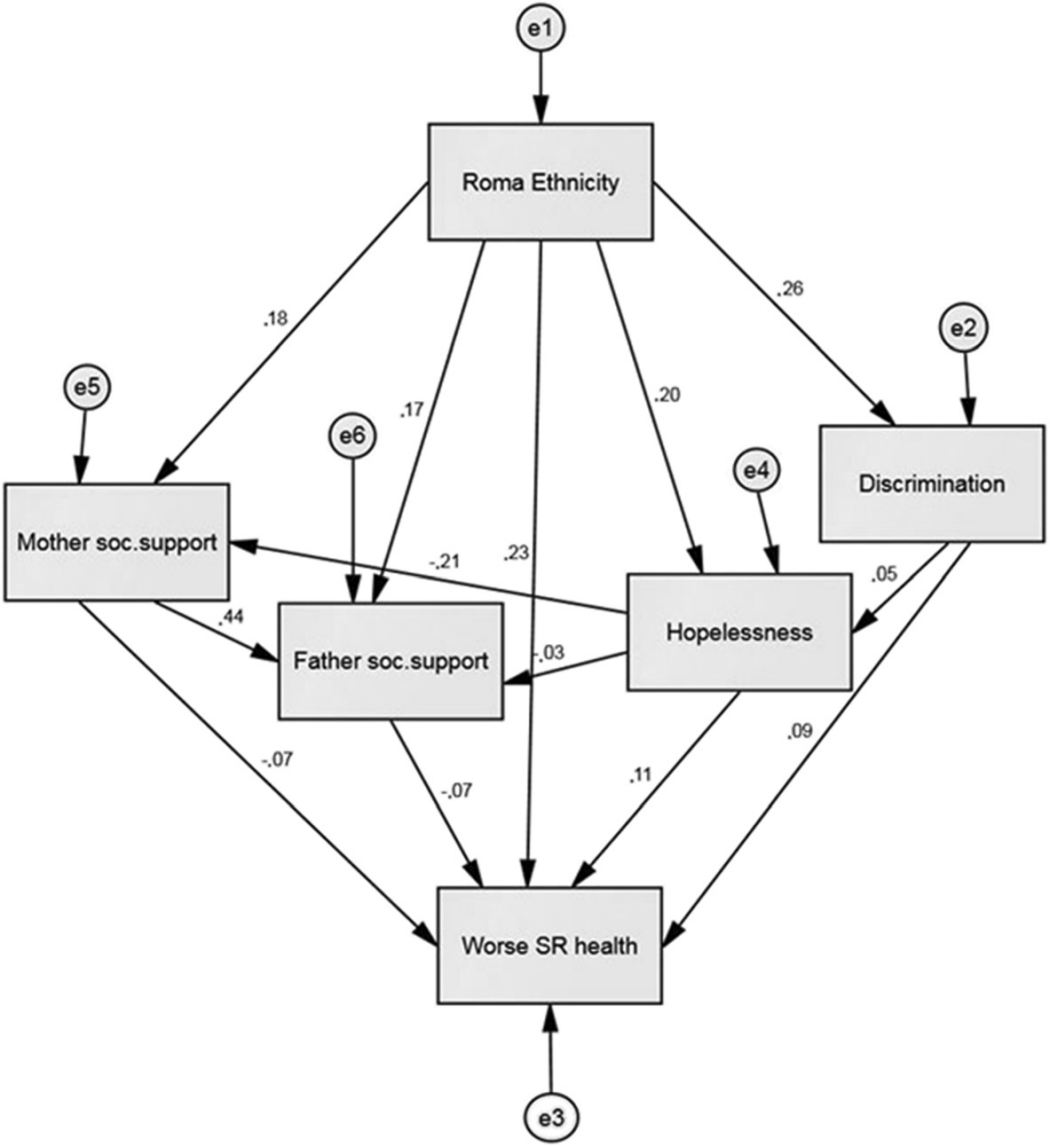
Structural model of the ethnicity effect on SRH mediated by discrimination, mother’s and father’s social support and hopelessness with indication of the association among variables using standardized coefficients. Note: e – represents residual variance

We assessed possible mediation of the above mentioned variables on the ethnicity-SRH effect by using the Sobel test. The Sobel test confirmed that discrimination, hopelessness and mother’s and father social support all were significant mediators of the ethnicity-SRH association (Sobel test values were 4.38***; 4.57***, -2.55** and -2.15*, respectively). Structural equation modelling also confirmed mediation and showed it in a more sophisticated way than simple Sobel tests. A part of the ethnicity effect on SRH was occurring via discrimination, mother’s and father’s social support, and hopelessness with a relatively small effect on SRH although SRH was more strongly influenced by the direct effect of ethnicity as shown in Fig. 1. Thus being Roma implies to have a worse SRH, but with higher perceived social support from mother and father the SRH might slightly improve. More hopelessness and discrimination on the other hand might slightly contribute to a worse SRH. The model also shows the effect of discrimination to be mediated through hopelessness on mother’s and father’s social support but the effect is rather small and almost trivial. The structural equation model yielded good fit as indicated by the goodness-of-fit indices (Chi-square = 4.062, p = 0.131; CMIN/DF = 2.031; CFI = 0.996; RMSEA = 0.033) (Fig. 1).

## Discussion

Our aim was to assess the possible mediating role of discrimination, mother’s and father’s social support and hopelessness in the ethnicity-SRH pathway and their contribution to differences in self-rated health between Roma and non-Roma adolescents. Further analysis revealed that discrimination, social support and hopelessness significantly mediated the ethnicity effect on SRH. Being discriminated against and having higher hopelessness consist a relatively great part of negative effect of ethnicity on SRH. Mother’s and father’s social support were also a part of the mediating pathway between ethnicity and SRH and constituted also a part of the pathway but in the opposite direction and social support can diminish the negative effect of discrimination and hopelessness.

Our results support previous findings that perceived discrimination is associated with poorer SRH [12–15]. However, from the simple association of discrimination with SRH we have also shown a more detailed picture enriched by the role of hopelessness and social support controlled for demographics and SES in the pathway between Roma ethnicity and SRH by testing of possible mediators. The mediating role of social support was already reported e.g. by Salonna et al. [45]. Hopelessness was not earlier tested before as a mediating variable in health studies.

Being Roma in our sample implies worse SRH, but this SRH is even worse when the Roma respondent reported being discriminated against. Similarly, feeling hopeless aggravated worse SRH even further. On the other hand, mother’s social support can compensate for the negative effect of ethnicity on health mediated by discrimination and hopelessness. The role of mother’s social support suggests that even the difficult living conditions represented by being Roma, living in a Roma settlement, being discriminated against and feeling hopeless, might by compensated for by a warm relationship with the mother [46]. The buffer effect of social support against the negative effect of perceived discrimination was reported by Ajrouch et al. [47]. What is interesting is that mother’s social support seems to be a more important factor than father’s social support in promoting better SRH.

Hopelessness, after discrimination, appeared to be a very influential factor related to adolescents’ SRH. The higher the hopelessness, the worse the SRH of the adolescent is. The connection of hopelessness with worse well-being and depression is already known [27, 29], and our study expands on the detrimental effect of hopelessness on SRH among adolescents who perceive discrimination, especially Roma adolescents, in whom the level of hopelessness and discrimination is much higher compared with non-Roma counterparts.

The worse SRH of Roma adolescents compared with their non-Roma counterparts might be partially attributed to their perceived discrimination and higher hopelessness, but these two factors do not explain the entire variability of their health. Another very important factor which affects their SRH might be the low education attainment of Roma parents and low SES widely prevalent among Roma living in settlements [8]. When evaluating the effect of several factors relevant for SRH, like discrimination, hopelessness and social support, parental education or other indicators of SES have to be taken into account, because, as our study has shown, every one of them plays an important role in the ethnicity–health relation. Also, there might be other factors related to Roma ethnicity, or their culture and habits, which might impact their health and which were not measured in our study and might confound associations, as parental education did.

### Strengths and limitations

Our study was conducted on a Roma sample, which is a hard-to-reach population. We succeeded in recruiting a considerable number of Roma adolescents. In addition, we achieved relatively high response rates in both samples of Roma and non-Roma. Due to the selection of participants with non-missing variables, the sample size was reduced, but the size was still large enough to perform all analyses with no impact on validity.

Besides these strengths, our study also has some limitations. A major limitation of our study may be the different methods of data collection among the Roma and non-Roma samples (interviews vs. questionnaires). We chose these different methods of data collection because in research comparing hard-to-reach groups with other groups, the use of different methodological approaches is sometimes unavoidable; see e.g. a recent study of Crone et al. [48] among ethnic minorities in The Netherlands. Furthermore, feasibility played a role, i.e. the acceptability for the target group and the available resources. It is likely that differences occurred in responses due to different methods of data collection, but Brittingham et al. [49] concluded that such differences tend to be small. The different approach to collect data from Roma than from non-Roma adolescents could have led to higher levels of social desirability among Roma, as disclosure may be lower in an interview [50–52]. Fortunately, we were able to adjust for this, but we cannot exclude some remaining information bias. Another limitation might be the use of a single item measure of perceived discrimination, which may have increased measurement error. However, previous research has shown this to be a valid measure of discrimination [53]. Moreover, its brevity led to a very small item-nonresponse, also increasing the validity of our approach. Finally, the low internal consistency of the social desirability scale (SDRS-5) might be considered as a limitation. The consequences of the low internal consistency of this scale are larger measurement errors and underestimation of its confounding effect.

Discrimination among Roma is a frequently discussed topic without objective and valid data. Our study brings fresh insights and an assessment of perceived discrimination among Roma adolescents compared with non-Roma adolescents.

### Implications

Since we found the worse self-rated health among Roma adolescents in comparison with the non-Roma population might be partially explained by higher exposure to perceived discrimination and hopelessness, interventions aiming to counteract such discrimination are justified. One place to start could be balancing the negative image of Roma in media with more positive ones and with education of the non-Roma population about the Roma with intention of replacing various stereotypes, superstitions and myths. Roma adolescents also reported having strong parental support with a protective effect on their self-rated health. This should be maintained and developed in cultural frameworks of this ethnic minority group.

Roma may avoid health services because they have experienced or heard about discrimination in health care settings [54]. A few negative interactions can be amplified in the community, as experiences are told and retold to others [6]. Wider utilisation of the existing Roma health assistants’ (or mediators’) program may be an effective route to prevent this but the project has been established in 2002 but have been used very scarcely until now [55]. E.g. in Slovakia only 0.6 Roma health assistants per 10,000 Roma are employed [56]. Further research on the effectiveness of the application of Roma health assistants’ program is urgently needed. Their work may also provide a means to realise a more positive image of Roma to counteract discrimination and improve the self-rated health of the Roma.

## Conclusions

Roma ethnicity, perceived discrimination, social support and hopelessness were the main predictors of poor self-rated health even after controlling for parental education and social desirability. As anticipated, perceived discrimination, like hopelessness, is a contributing factor for poor self-rated health. Parental social support also partially contributes to the effect on SRH, but in the opposite direction than discrimination and hopelessness, and thus protects against poor SRH. Perceived discrimination, hopelessness and mother’s and father’s social support are mediators of the ethnicity-health association. Our study presents one of the first findings about discrimination and health among Roma adolescents and explores potential protective and risk factors in such an association, providing important clues to improving their health.

## Abbreviations

EU-MIDIS: European Union minorities and discrimination survey
SES: Socioeconomic status
SRH: Self-rated health
ISRD: International Self-Report Delinquency Study
SDRS-5: Social Desirability Response Set
OR: Odds ratio
CI: Confidence interval
